# Association between the 20-minute whole blood clotting test and fibrinogen concentrations in green pit viper envenomations in Bangkok

**DOI:** 10.1371/journal.pntd.0014121

**Published:** 2026-03-16

**Authors:** Supa Niruntarai, Sivilai Hengtrakul, Rittirak Othong

**Affiliations:** 1 Department of Emergency Medicine, Faculty of Medicine Vajira Hospital, Navamindradhiraj University, Dusit, Bangkok, Thailand; 2 Department of Emergency Medicine, Taksin Hospital, Bangkok, Thailand; University of Liverpool, UNITED KINGDOM OF GREAT BRITAIN AND NORTHERN IRELAND

## Abstract

**Background:**

The 20-minute whole blood clotting test (20WBCT) is widely used in patients with viper envenomation in resource-limited settings. The unclotted result guides the need for antivenom administration. Confusion has arisen on how to interpret “partially clotted” for the test due to a paucity of data. This study’s primary aim was to evaluate the association between fibrinogen concentrations (FC) and states of clotting from the 20WBCT in green pit viper (GPV) envenomation.

**Methods:**

Patients aged ≥18 years who presented to our hospital with GPV bites were enrolled between September 2022 and November 2023. All 20WBCT were done by laboratory technicians and the results were video recorded. Corresponding blood samples were quantified for FC. Two investigators, blinded to clinical/laboratory data, interpreted clotting results from recordings. A third investigator resolved conflicts.

**Results:**

Thirty-nine patients contributed 188 blood samples. 20WBCT results were categorized as completely clotted, partially clotted (subdivided into mostly or minimally clotted), or unclotted, with median FC of 259.1, 223.5, 226.1, and 48.7 mg/dL, respectively. FC in the unclotted group were significantly lower than those in the other groups (completely: p = 0.001; mostly: p < 0.001; minimally: p = 0.002), with no differences among the completely clotted and two subgroups of the partially clotted. The unclotted 20WBCT had 28.6% sensitivity and 99.4% specificity for detecting FC < 100 mg/dL. Further analysis found that a FC < 70 mg/dL best predicted the unclotted result (the area under the receiver operating characteristic curve was 0.99 (0.99-1.0) and the accuracy was 98.9%).

**Conclusions:**

Compared with the partially clotted samples (either mostly or minimally clotted), FC were significantly lower in the unclotted, but similar to the completely clotted samples. The partially clotted samples should be interpreted as completely clotted.

## Author summary

Green pit viper envenomation can cause coagulopathy. The 20-minute whole blood clotting test (20WBCT) is used to guide antivenom treatment in a large number of hospitals in developing nations. However, it is unclear how to interpret a “partially clotted” result. We studied patients with green pit viper bites, comparing the 20WBCT results with their fibrinogen concentrations. We found that “unclotted” samples had very low fibrinogen concentrations, while the concentrations in “partially clotted” blood were similar to those in “completely clotted” samples. These findings suggest that “partially clotted” results should be interpreted as “clotted.” While fibrinogen assays are the “reference standard” for diagnosing venom-induced consumptive coagulopathy, their reliance on specialized equipment and cold-chain reagents limits their utility in rural areas where most viper envenomations occur. The 20WBCT remains the primary diagnostic tool in these resource-limited settings. By refining the interpretation of this test—specifically by simplifying the binary ‘clot versus no-clot’ decision—our study directly addresses a critical gap in public health infrastructure, providing clinicians with a more reliable method to make life-saving antivenom decisions at the bedside.

## Introduction

Snakebite represents a health burden across many tropical and subtropical nations, and it was recognized by the World Health Organization (WHO) as a neglected tropical disease in 2017. Annually, as many as 2 million people in Asia experience snakebite envenomation, while in Africa, approximately 500,000 snakebite cases necessitate treatment. Although antivenom remains the mainstay of therapy, limited production and challenges in assessing its quality and appropriateness persist [1]. Furthermore, significant knowledge gaps regarding snakebite envenomation underscore the need for evidence-based approaches to treatment and investigation.

In Thailand, snakebites also pose a major threat to public health. It was estimated that 8,525–8,906 snakebites occurred each year in the country, with 3,766–6,482 cases requiring antivenom, 2–7 deaths, and up to 7 amputations [2]. Green pit vipers (GPV) are commonly found in multiple regions in Thailand, accounting for 31.9% of all venomous snakebites [3,4]. In Bangkok, *Trimeresurus albolabris* and *T. macrops* are the two species of green pit vipers found [5], and they are responsible for over 90% of all venomous snakebites [6].

*T. albolabris* (the white-lipped pit viper) is not only distributed throughout several regions of Thailand, but it is also found across South and Southeast Asia, including northeastern India, Bangladesh, Nepal, Myanmar, Malaysia, Cambodia, Laos, and Vietnam, as well as parts of East Asia such as southern China, Taiwan, and Hong Kong [7–9]. Similarly, *T. macrops* (the big-eyed pit viper) can be found in Cambodia, Laos, and Vietnam [10].

The venom of the green pit vipers (GPV) is hematotoxic, containing a thrombin-like enzyme that plays a crucial role in accelerating the conversion of fibrinogen into fibrin. However, the fibrin clots formed through this process are abnormal, friable, and loose. These abnormal clots cannot function properly and are easily eliminated from the body. This venom-induced phenomenon leads to a significant decrease in fibrinogen concentrations in the blood, causing hypofibrinogenemia. Additionally, the venom triggers the breakdown of blood clots, known as hyperfibrinolysis, resulting in elevated concentrations of fibrinogen degradation products (FDPs) [11–13].

Fibrinogen concentrations of less than 100 mg/dL have been used as a cut-off value to indicate the need for antivenom administration for severe GPV envenomation due to the risk of hemorrhage [14,15]. Nevertheless, quantifying fibrinogen concentrations is quite limited and may not be accessible in a large number of hospitals, especially those in developing countries [16]. In hospitals where fibrinogen concentration quantifications cannot be performed, the 20-minute whole blood clotting test (20WBCT) is often used instead.

The 20-minute whole blood clotting test (20WBCT) has been recommended by the World Health Organization (WHO) as a bedside diagnostic tool for venom-induced consumptive coagulopathy (VICC) worldwide especially in developing countries [17] because of its ease of use, accessibility, and minimal resource requirements [18]. Typically, the 20WBCT results are interpreted as either “clotted” (normal) or “unclotted” (abnormal), and for the abnormal result, this indicates the need for antivenom administration. In clinical practice the results of the 20WBCT are not always binary as clotted or unclotted, “partially clotted” is a commonly encountered finding. Partially clotted samples have been found to be problematic in 18.2% of those who answered to a survey study on variation of practice in WBCT interpretation [19]. For samples with partial clot formation, it was found that some blood samples do not form a clot completely at the 20-minute mark. Both solid clots and liquid blood is seen at the same time during tilting the tubes at 45 degrees. A study on Malayan pit viper envenomation revealed significant differences in fibrinogen concentrations among the three states of 20WBCT results (“clotted,” “partially clotted,” and “unclotted”) [20]. It remains unclear how to properly interpret a sample with a partial clot, whether it is normal or abnormal. This ambiguity could critically affect the decision to prescribe antivenom to those with snakebite envenomation, particularly in resource poor settings where antivenom is in limited supply.

The primary objective of this study was to determine the association between fibrinogen concentrations and the clotting states of the 20WBCT (unclotted, partially clotted, and completely clotted) in patients envenomated by green pit vipers. In addition, diagnostic parameters (sensitivity, specificity, and accuracy) of each clotting state were also evaluated, using the cut-off value for hypofibrinogenemia below 100 mg/dL.

## Methods

### Study design

This was a cross-sectional diagnostic study conducted at the Emergency Medicine Department (ED) and the Central Laboratory Department of the Faculty of Medicine Vajira Hospital, a tertiary university hospital in Bangkok. The study was approved by the Faculty of Medicine Vajira Hospital’s Institutional Review Board (COA 164/2565).

### Study participants

We prospectively enrolled all eligible patients aged 18 years or older who presented to the ED due to a GPV bite between 1 September 2022 and 30 November 2023. The diagnosis of a GPV bite was made based on one of the following three criteria: 1) The GPV snake was brought to the ED or a photograph of the snake was taken for identification; 2) The patient described the snake as having a green body with a red-brown tail; 3) In cases where the patient was bitten by a green snake but the color of the tail was not observed, and two fang marks were present along with swelling involving more than one major joint, or along with any systemic effects including venous clotting time > 20 minutes, unclotted 20WBCT, INR ≥ 1.2, platelet count < 100,000/mm³, fibrinogen concentration <100 mg/dL, or systemic bleeding, and without signs of muscle weakness [21]. Patients were excluded if they had a pre-existing history of coagulation abnormalities, including hemophilia, Von Willebrand disease, hypo-prothrombinemia, or cirrhosis with an INR greater than 1.4, or if they were currently prescribed anticoagulant medications. We did not exclude patients on antiplatelet therapy because Othong et al. previously demonstrated that antiplatelet use is not associated with prolonged VCT in green pit viper envenomation (Fisher’s exact test) [22]. Patients who were unable to communicate in Thai were also excluded. All participants provided written informed consent prior to study enrollment.

### Study protocol

In eligible participants, blood samples were drawn for complete blood count (CBC), prothrombin time (PT), activated partial thromboplastin time (aPTT), international normalized ratio (INR), venous clotting time (VCT), thrombin time (TT), fibrinogen concentration, and 20WBCT at 2, 5, 24 ± 12, 48 ± 12, and 72 ± 12 hours after the bite. All patients with GPV bites were directed to the Central Laboratory Department for blood collection. Coagulation tests, including the 20WBCT, were performed concurrently using a single blood draw. The 20WBCT was conducted by trained laboratory technicians. If a patient received GPV antivenom, these blood tests were repeated every 6–12 hours until resolution of coagulopathy.

For the 20WBCT, hospital laboratory technicians performed the test in the Central Laboratory Department by placing 2 mL of blood into a clean and dry 13 (outer diameter) × 100 (length) mm. glass tube, leaving it still for 20 minutes in a temperature-controlled laboratory environment (approximately 25°C). After 20 minutes, a laboratory technician raised and tilted the tube from an upright position (90°) to a 45–60° angle while recording a video during the tilting process for further interpretation by investigators [23].

In this study, the 20WBCT results were visually interpreted into three states:

- Completely clotted: The entire blood sample in the tube becomes a solid clot that adheres to the glass tube upon tilting.
- Partially clotted: A blood clot is observed at the bottom of the glass tube along with liquid blood that fails to cling to the tube, further subdivided into:
  - Mostly clotted: The clot is observed and the size is estimated to be larger than 50% of the total blood volume in the tube.
  - Minimally clotted: The clot is observed and the size is estimated to be smaller than 50% of the total blood volume in the tube.
- Unclotted: The entire blood sample remains liquid, with no clot formation is seen.

Two investigators independently reviewed and interpreted the 20WBCT recordings. In cases of disagreement, a third investigator (a senior Medical Toxicologist) reviewed the recordings, and the final result was determined by agreement of two out of three investigators. If all three interpretations differed, a consensus discussion was held to reach a final decision. All investigators were blinded to participants’ clinical data as well as fibrinogen concentrations, and other laboratory results during the interpretation of the 20WBCT to prevent bias.

Analyses of fibrinogen concentrations, PT, PTT, TT were performed in the hospital central laboratory using the Sysmex CS-2500 automated coagulation analyzer. For the purpose of data analysis, cut-off values for PT, PTT, and TT were defined as the upper limits of the normal laboratory reference ranges.

For the VCT, details on the method of performing are available elsewhere [24]. Two common cut-off values have been used, > 20 minutes and > 30 minutes, with different sensitivities and specificities [15,16].

Blood samples without the 20WBCT or corresponding fibrinogen concentrations were excluded from data analysis.

### Sample size calculation

This study compared fibrinogen concentrations among patients categorized by clotting states of 20WBCT into clotted, partially clotted, and unclotted groups. Sample size was estimated using G*Power version 3.1.9.4 (Heinrich Heine University Düsseldorf, Düsseldorf, Germany) with one-way ANOVA, based on data from Thongtonyong and Chinthammitr [20], who reported median fibrinogen concentrations of 193 mg/dL (31–451), 153 mg/dL (34–426), and 31 mg/dL (31–330) for the clotted, partially clotted, and unclotted 20WBCT groups, respectively. Medians and ranges were converted to means and standard deviations using the Hozo’s method [25], yielding 217.0 ± 105.0 mg/dL, 191.5 ± 98.0 mg/dL, and 105.8 ± 75.7 mg/dL, respectively.

The effect size was 0.55. With α = 0.05, power = 0.90, and three groups, the minimum required sample size was 45. Allowing for repeated sampling and a 10% dropout rate, the final target was set at 50 blood samples.

### Statistical analysis

Demographic, clinical, and laboratory parameters were described for the study population. Continuous variables were expressed as a mean with a standard deviation (SD) for normally distributed data, and as a median with an interquartile range (IQR) for non-normally distributed data. Categorical variables were presented as frequency and percentages. Differences in fibrinogen concentrations among the clotting states of the 20WBCT were analyzed using generalized estimating equations (GEE) with a linear model and a population-averaged approach. Sensitivity, specificity, and the area under the receiver operating characteristic curve (AUROC) of each clotting state and each coagulation test were calculated against fibrinogen concentrations below 100 mg/dL. The optimal cut-off point for the INR was determined using the Liu method (Youden index) [26]. All *p*-values reported are two-sided, with statistical significance defined as *p* < 0.05. All analyses were performed using Stata version 18 (StataCorp, College Station, TX, USA).

## Results

During the study period, 40 patients with GPV bites were screened, and one individual was excluded due to inability to communicate. Finally, 39 patients were enrolled in the study. A total of 189 blood samples were collected for coagulation testing. Of these, one sample was excluded because the bottom of the tube was not visible in the video recording, and it was considered uninterpretable as to whether it was unclotted or minimally clotted.

Demographic data are shown in Table 1. Most patients were male (59%), with a mean age of 57.6 years. Comorbidities were present in 38.5% of patients, with hypertension being the most common. Five patients (12.8%) were taking antiplatelet medications (two on aspirin, two on cilostazol, and one on ticagrelor). The median elapsed time from bite to the ED was 55 minutes (IQR: 30–105 minutes). Fingers (33.3%) and feet (33.3%) were the most common bite sites.

**Table 1:** Demographic data.

Characteristics	Total (n = 39)
**Age in years, mean (± SD)**	57.6 ± 15.3
**Sex**	
**Male, n (%)**	23 (59.0)
**Underlying, n (%)**	
**Hypertension**	12 (30.8)
**Diabetes mellitus**	6 (15.4)
**No underlying diseases**	24 (61.5)
**Elapsed time from bite to the ED (minutes), median (IQR)**	55 (30-105)
**Bite site, n (%)**	
**Fingers**	13 (33.3)
**Hands**	7 (17.9)
**Toes**	6 (15.4)
**Feet**	13 (33.3)
**Grade of edema at ED visit (grade 0–7), n (%)**	
**Grade 0: no edema**	1 (2.6)
**Grade 1: local edema**	12 (30.8)
**Grade 2: up to one major joint**	6 (15.4)
**Grade 3: above one major joint**	10 (25.6)
**Grade 4: up to two major joints**	7 (17.9)
**Grade 5: above two major joints**	2 (5.1)
**Grade 6: impending compartment syndrome**	1 (2.6)
**Grade 7: compartment syndrome**	0 (0)
**Pain score (Numeric Rating Scale 0–10)**	
**First ED visit, median (IQR)**	5 (0-6)
**Maximum pain score, median (IQR)**	5 (2–6)
**Other types of local effects, n (%)**	
**Ecchymosis**	0 (0)
**Blister**	0 (0)
**Necrosis**	0 (0)
**Wound infection (cellulitis)**	4 (10.3)
**Systemic effects**	
**Abnormal bleeding**	0 (0)
**Severe hypersensitivity reactions from venom**	0 (0)
**Indication for antivenom treatment, n (%)**	9 (23.1)
**Hypofibrinogenemia**	6 (15.4)
**Prolonged VCT**	1 (2.6)
**Thrombocytopenia**	1 (2.6)
**Impending compartment syndrome**	1 (2.6)
**Adverse reactions from antivenom, n (%)**	
**Mild hypersensitivity reactions**	0 (0)
**Severe hypersensitivity reactions**	1 (2.6)

Most cases presented with grade 1 local edema (30.8%) and with a median pain score of 5 at the initial ED visits. One patient developed impending compartment syndrome, and four patients developed cellulitis. No cases of systemic bleeding or death were observed.

A total of nine patients (23.1%) received antivenom (Table 1), seven patients for coagulopathy (six with low fibrinogen concentrations and one with prolonged VCT), another one for thrombocytopenia (fibrinogen concentration 329.5 mg/dL and platelet count 92,000 cu.mm.), and the last one for impending compartment syndrome. One patient developed a severe hypersensitivity reaction following antivenom administration.

**Fig 1:**
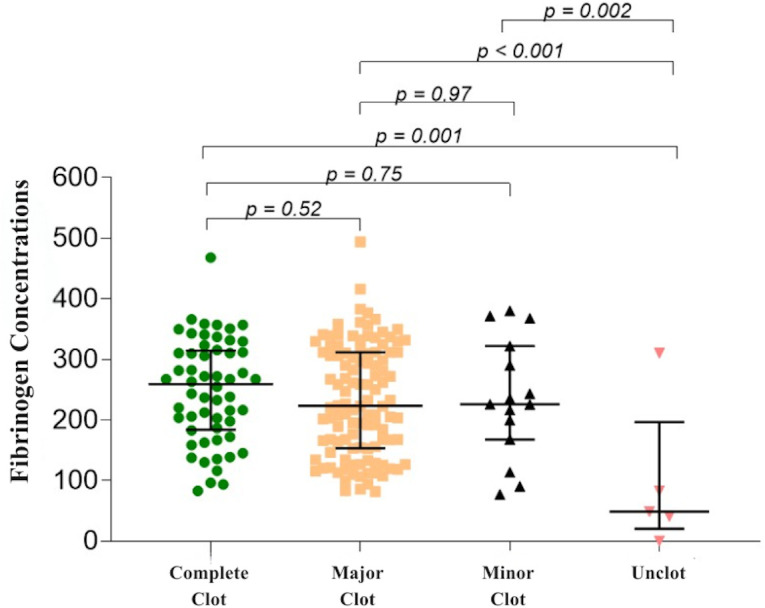
Dot plots comparing median fibrinogen concentrations among the states of 20WBCT (complete clot, major clot, minor clot, and unclot). *P-value was evaluated by Generalized Estimating Equations with linear model and population-averaged model.

Table 2 shows the median fibrinogen concentrations in each clotting state of the 20WBCT. We observed a significant difference between unclotted samples and all other groups (with completely clotted: *p* = 0.001, mostly clotted: *p* < 0.001, minimally clotted: *p* = 0.002). There were no significant differences among the completely clotted, mostly clotted, and minimally clotted groups, as illustrated in the dot plot (Fig 1). However, in the completely clotted samples, 5.4% (3/56) had fibrinogen concentrations less than 100 mg/dL (82.9, 93.7, and 96.3 mg/dL). For the samples having major clot, 5 out of 112 samples (4.5%) revealed fibrinogen concentrations below 100 mg/dL (81.9, 83.3, 85.7, 93.9, and 99.7 mg/dL). In the samples with minor clot, 13.3% (2/15) had low fibrinogen concentrations (77.0 and 90.6 mg/dL).

**Table 2:** Median fibrinogen concentrations in each state of the 20WBCT.

20WBCT	N (%)	Fibrinogen concentration (mg/dL) Median (IQR)	Overall P-value
**Complete clot**	56 (29.8)	259.1 (184.8-313.7)	0.001
**Partial clot**	127 (67.5)	225.6 (154.1-311.7)	
**Unclot**	5 (2.7)	48.7 (41.0-82.9)	
**Complete clot**	56 (29.8)	259.1 (184.8-313.7)	0.004
**Major clot**	112 (59.5)	223.5 (153.7-311.7)	
**Minor clot**	15 (8.0)	226.1 (167.8-322.1)	
**Unclot**	5 (2.7)	48.7 (41.0-82.9)	

Sensitivity, specificity, area under the receiver operating characteristic curve (AUROC), and accuracy for 20WBCT, VCT, INR, PT, aPTT, and TT were summarized in Table 3. Using a fibrinogen concentration of <100 mg/dL as the cut-off value for hypofibrinogenemia, the unclotted 20WBCT demonstrated a sensitivity of 28.6% and a specificity of 99.4%, with an AUROC of 0.64 (S1 Fig). When interpreting both the unclotted and minimally clotted categories together as an abnormal result, the sensitivity increased to 42.9%, but the specificity decreased to 90.9%. The unclotted 20WBCT alone had the highest diagnostic accuracy at 94.2%, followed by the venous clotting time (VCT) ≥ 30 minutes and the thrombin time (TT) > 20 seconds. Among the coagulation tests, an INR ≥ 1.015 showed the highest AUROC at 0.82.

**Table 3:** Performance of different coagulation tests against fibrinogen concentration < 100 mg/dL.

	Fibrinogen concentration < 100 mg/dL			
	Sensitivity (%) (95% CI)	Specificity (%) (95% CI)	AUROC (%) (95% CI)	Accuracy (%)
20WBCT				
Unclot	28.6 (8.4-58.1)	99.4 (96.9-100)	0.64 (0.52-0.76)	94.2
Unclot+minimal clot	42.9 (17.7-71.1)	90.9 (85.7-94.7)	0.67 (0.53-80.5)	87.4
VCT				
> 20 min	28.6 (8.4-58.1)	96.0 (91.9-98.4)	0.62 (0.50-0.75)	90.5
≥ 30 min	21.4 (4.7-50.8)	100 (97.9-100)	0.61 (0.50-0.72)	93.7
INR ≥ 1.2	25.0 (5.5-57.2)	97.1 (93.4-99.1)	0.61 (0.48-0.74)	90.0
INR ≥ 1.015*	92.9 (66.1-99.8)	70.5 (63.1-77.1)	0.82 (0.74-0.89)	72.1
PT > 13.5 s**	35.7 (12.8-64.9)	93.8 (89.1-96.8)	0.65 (0.52-0.77)	89.5
PTT > 29.6 s**	0 (0.0-26.5)	96.0 (91.8-98.4)	0.48 (0.50-0.49)	87.4
TT > 20 s**	33.3 (9.9-65.1)	100 (97.9-100)	0.67 (0.53-0.81)	92.1

* Optimal cut-off point selected using Youden index, min = minutes, s = seconds.

** These cut-off values were from the central laboratory’s references.

Because there have been no consensus cut-off values of fibrinogen concentration for diagnosing hypofibrinogenemia in snakebite envenomation, we further lowered the fibrinogen cut-off value to < 70 mg/dL resulting in even higher sensitivity and AUROC for almost all coagulation tests, except for INR ≥ 1.015 (Table 4 and S2 Fig). Additionally, when exploring fibrinogen cut-off concentrations of < 60 mg/dL and < 50 mg/dL, we found that sensitivity, specificity, and AUROC remained comparable to those observed at the < 70 mg/dL threshold.

**Table 4:** Performance of coagulation tests against fibrinogen concentration < 70 mg/dL.

	Fibrinogen concentration < 70 mg/dL			
	Sensitivity (%) (95% CI)	Specificity (%) (95% CI)	AUROC (95% CI)	Accuracy (%)
20WBCT				
Unclot	100 (29.2-100)	98.9 (96.2-99.9)	0.99 (0.99-1.00)	98.9
Unclot+minimal clot	100 (29.2-100)	89.8 (84.6-93.8)	0.95 (0.93-0.97)	90.0
VCT				
> 20 min	100 (29.2-100)	95.7 (91.7-98.1)	0.98 (0.96-0.99)	95.3
≥ 30 min	100 (29.2-100)	100 (98.0-100)	1.00 (1.00-1.00)	99.5
INR ≥ 1.2	100 (15.8-100)	96.7 (93.0-98.8)	0.98 (0.97-0.99)	94.2
INR ≥ 1.015*	100 (29.2-100)	66.8 (59.6-73.5)	0.83 (0.80-0.87)	67.4
PT > 13.5 s**	100 (29.2-100)	93 (88.4-96.2)	0.97 (0.95-0.98)	93.2
PTT > 29.6 s**	0 (0-84.2)	96.2 (92.3- 98.4)	0.48 (0.47-0.50)	95.1
TT > 20 s**	100 (15.8-100)	98.9 (96.1-99.9)	0.99 (0.99-1.00)	98.9

* Optimal cut-off point selected using Youden index, min = minutes, s = seconds.

** These cut-off values were from the central laboratory’s references.

## Discussion

This study primarily investigated the correlation between each state of 20WBCT (unclotted, partially clotted (mostly or minimally clotted), and completely clotted) results and fibrinogen concentrations in GPV envenomation, which has not been previously done for this species. The results were consistent with a very few studies previously conducted in patients envenomated by other species, *Bothrops* spp. and Malayan pit vipers in terms of a similar relationship between the unclotted 20WBCT and low plasma fibrinogen concentrations [20,27]. There was a statistically significant difference among the unclotted, partially clotted, and completely clotted groups (Table 2), similar to findings in a study on Malayan pit viper envenomation. However, the median fibrinogen concentrations in all three 20WBCT groups in our study were higher: 48.7 *VS* 31 mg/dL, 225.6 *VS* 153 mg/dL, and 259.1 *VS* 193 mg/dL in the unclotted, partially clotted, and completely clotted groups, respectively [20]. These differences might be attributed to variations in venom composition between the two species, leading to different intensity on the coagulation system [28,29], as well as differences in methods and interpretation of the 20WBCT between studies. Additionally, we attempted to further subclassify the partial clot into ‘major clot’ or ‘minor clot’ and found no statistically significant differences in fibrinogen concentrations between these two groups (Fig 1). Fibrinogen concentrations in the completely clotted samples were also not significantly different from those in the mostly clotted or minimally clotted samples (Fig 1). As a result, we suggest that clinicians interpret all partially clotted 20WBCT samples, whether major or minor, as if they were completely clotted. When both partial and complete clots were categorized as negative results and only an unclotted result was interpreted as positive, we calculated the inter-rater reliability between the two observers, and we found that the inter-rater reliability demonstrated perfect agreement (Cohen’s kappa = 1.00). Both raters achieved 100% concordance in identifying positive and negative outcomes. While discrepancies occurred in distinguishing between complete, major, and minor clots (complete vs. major, n = 11; major vs. minor, n = 19), these differences did not affect clinical management, as all degrees of partial clotting were ultimately managed as negative (complete) outcomes.

In this study, six individuals exhibited severe hypofibrinogenemia (< 100 mg/dL) and received antivenom. Only one of them also had thrombocytopenia. None manifested systemic bleeding. Other green pit viper envenomation studies have reported very few cases of systemic bleeding, despite all of them having only minor bleeding. None experienced life-threatening hemorrhage or death [15,16]. Among five samples from three patients with unclotted 20WBCT results, fibrinogen concentrations were 0, 41.0, and 82.9 mg/dL in the first patient; 48.7 mg/dL in the second; and 310.3 mg/dL in the third. Two patients received antivenom because of hypofibrinogenemia.

One study reported that antivenom administration can cause early adverse reactions (EARs) in more than 10% of cases, with severe EARs such as hypoxia, hypotension, and neurological compromise occurring at rates of 0.3% with GPV antivenom and 3.4% with hemato-polyvalent antivenom [30]. Given the risk of severe EARs and antivenom being in limited supply in some places, antivenom should be administered only when clearly necessary.

We questioned whether, in case of isolated hypofibrinogenemia without thrombocytopenia, the current fibrinogen threshold of <100 mg/dL for initiating antivenom treatment would be appropriate and whether an alternative threshold should be considered. According to current guidelines, a fibrinogen concentration below 100 mg/dL indicates significant hypofibrinogenemia [15]. Most studies with this threshold primarily were done on patients with trauma or massive hemorrhage [14,31]. In the context of perioperative transfusion, treatment initiation is generally recommended when fibrinogen concentrations fall below 80–100 mg/dL [32]. Notably, patients with trauma or surgical bleeding exhibit a different pathophysiology compared to those envenomated by vipers, as those surgical or trauma cases typically have wounds with cut vessels and raw tissue surfaces which make them much more susceptible to bleeding and they would need higher fibrinogen concentrations to maintain hemostasis. In contrast, snakebite patients usually only have two tiny puncture wounds. Peyvandi et al. conducted a study on patients with fibrinogen deficiency and found that a mean fibrinogen activity level of at least 73 mg/dL was protective against spontaneous hemorrhage [33]. Some other studies investigating hypofibrinogenemia unrelated to trauma suggest initiating treatment when fibrinogen concentrations fall below 50 mg/dL [34,35].

In a systematic review and meta-analysis by Lamb et al., the pooled weighted sensitivity and specificity of the 20WBCT for detecting fibrinogen concentrations <100 mg/dL were 0.72 (95% CI, 0.58–0.83) and 0.94 (95% CI, 0.88–0.98), respectively, with a summary receiver operating characteristic (SROC) AUC of 0.93 (0.91–0.95). When the fibrinogen threshold was lowered to <50 mg/dL (or INR > 5), sensitivity increased notably to 0.91 (95% CI, 0.64–0.98), with a corresponding SROC AUC of 0.96 (0.94–0.97) [36].

Consequently, we evaluated alternative fibrinogen thresholds of <70, < 60, and <50 mg/dL. We found that a cut-off value of 70 mg/dL raised the sensitivity, specificity, accuracy, and AUROC of nearly all coagulation tests—including the 20WBCT—to 100% or near 100%, with the exception of aPTT (Table 4 and S2 Fig). For thresholds of 60 and 50 mg/dL, the diagnostic parameters remained identical to those observed at the 70 mg/dL level. Furthermore, among samples displaying complete, major, or minor clots on the 20WBCT, only 5.4%, 4.5%, and 13.3%, respectively, had fibrinogen concentrations between 70 and 100 mg/dL; notably, none of these patients experienced systemic bleeding. These findings suggest that a fibrinogen level greater than 70 mg/dL may be sufficiently safe for a watchful observation approach, indicating that antivenom might not be required. However, this 70 mg/dL threshold should be applied cautiously in cases of isolated hypofibrinogenemia, as concurrent thrombocytopenia is associated with an increased risk of hemorrhage (odds ratio 4.19, *p*-value 0.0005) [37]. In such instances, a higher fibrinogen threshold (100 mg/dL) might be a more appropriate indicator for antivenom administration. While the study by Lamb et al. showed that lowering the fibrinogen threshold from 100 to 50 mg/dL increased the sensitivity and SROC AUC of the 20WBCT, it did not account for the potential complications of a lower threshold or the additive risk of concurrent thrombocytopenia [36]. However, the proposed 70 mg/dL threshold in our study is limited by the small number of non-clotting blood samples; therefore, further research is warranted to validate the optimal fibrinogen threshold for antivenom treatment.

### Limitations

We prospectively collected almost 200 blood samples but only a small number of these samples had fibrinogen concentrations below 100 mg/dL and were unclotted. These factors might influence the sensitivity estimates and should be considered when interpreting the results. Future studies with a larger number of cases with severe hypofibrinogenemia are needed to provide more robust estimates of diagnostic performance.

There were small variations in blood volumes collected for the 20WBCT, despite our attempts to control the method blood was collected by having laboratory technicians collect blood samples for the study using standard procedures. They were also briefed on how and how much blood was needed per tube.

This study was done in those who were bitten only by green pit vipers, results might be different with different species.

## Conclusions

Our study demonstrated that fibrinogen concentrations in the unclotted 20WBCT group were significantly lower than those in the partially clotted and completely clotted groups. Further subdivision of the partially clotted group into mostly and minimally clotted categories revealed no statistically significant differences. Based on these findings, we would interpret all partially clotted results—whether minimally or mostly clotted—as clotted, and reserve antivenom treatment only for patients with an unclotted 20WBCT. Given the small number of cases with severe hypofibrinogenemia in this cohort, larger studies are needed to validate these conclusions.

## Supporting information

S1 Fig AUROC of 20WBCT for predicting fibrinogen concentrations < 100 mg/dL. (PDF)

S2 Fig AUROC of 20WBCT for predicting fibrinogen concentrations < 70 mg/dL. (PDF)

S1 Table 20WBCT and Fibrinogen Concentrations at Serial Time Intervals of Blood Sampling. (DOCX)

S2 Table Raw data: Baseline characteristics. (XLSX)

S3 Table Raw data: Laboratory results. (XLSX)
