# Supplementary figures and images for: Association between the 20-minute whole blood clotting test and fibrinogen concentrations in green pit viper envenomations in Bangkok
Source: PLoS Negl Trop Dis. 2026 Mar 16;20(3):e0014121. doi: 10.1371/journal.pntd.0014121 (PMC13004501; doi:10.1371/journal.pntd.0014121)

S1 Fig. AUROC of 20WBCT for predicting fibrinogen concentrations < 100 mg/dL

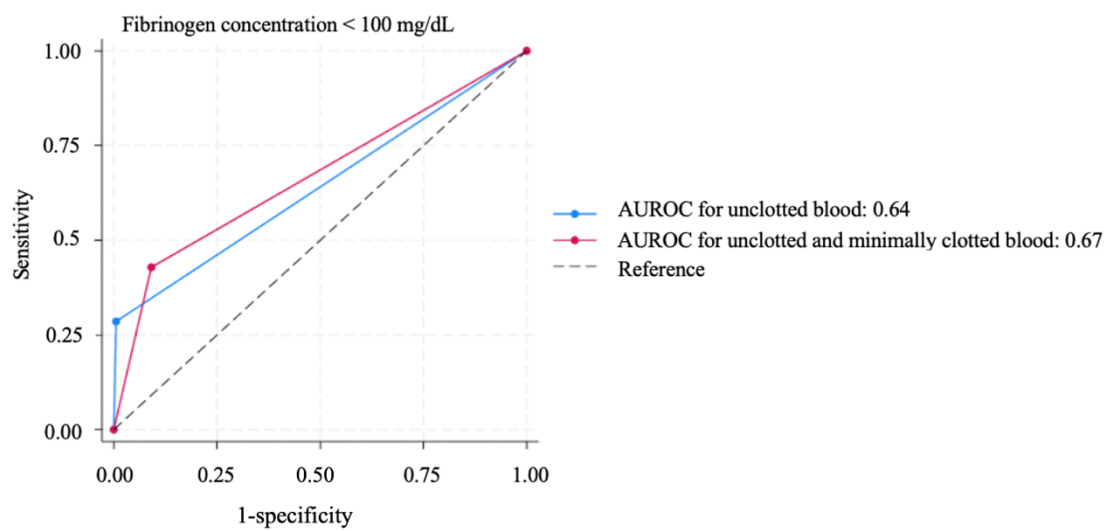

Supplement: S1 Fig — (PDF) [file pntd.0014121.s001.pdf]

S2 Fig. AUROC of 20WBCT for predicting fibrinogen concentrations < 70 mg/dL.

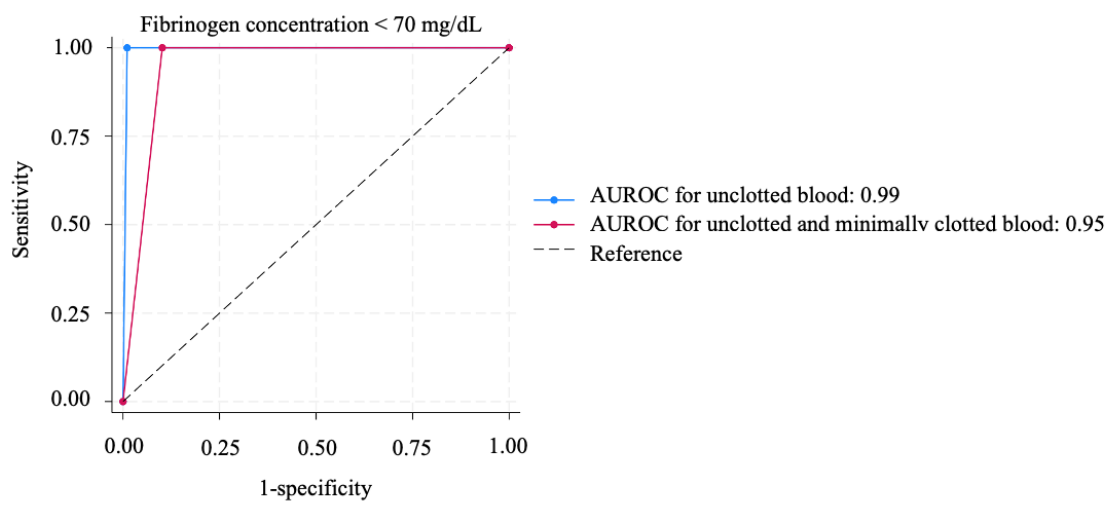

Supplement: S2 Fig — (PDF) [file pntd.0014121.s002.pdf]
