# Supplementary material for: Association between the 20-minute whole blood clotting test and fibrinogen concentrations in green pit viper envenomations in Bangkok
Source: PLoS Negl Trop Dis. 2026 Mar 16;20(3):e0014121. doi: 10.1371/journal.pntd.0014121 (PMC13004501; doi:10.1371/journal.pntd.0014121)
Supplement: S1 Table — (DOCX) [file pntd.0014121.s003.docx]

**S1 Table. 20WBCT and Fibrinogen Concentrations at Serial Time Intervals of Blood Sampling**

| **Case No.** | **Time (post-bite)** | **20WBCT** | **Fibrinogen (mg/dL)** |
| --- | --- | --- | --- |
| **1** | 2h | CC | 263.2 |
|  | 5h | PC | 267.5 |
|  | 24h | CC | 277.5 |
|  | 48h | PC | 258.9 |
| **2** | 2h | CC | 243.3 |
|  | 5h | CC | 198.0 |
|  | 24h | PC | 152.8 |
|  | 48h | CC | 166.6 |
|  | 72h | PC | 168.1 |
| **3** | 5h | PC | 208.4 |
|  | 24h | PC | 250.3 |
|  | 48h | PC | 216.9 |
|  | 72h | PC | 235.3 |
| **4** | 8h | PC | 258.6 |
|  | 13h | PC | 340.9 |
|  | 24h | PC | 340.9 |
|  | 48h | PC | 345.3 |
|  | 72h | CC | 358.5 |
|  | 96h | PC | 329.5 |
|  | 102h | PC | 332.7 |
|  | 108h | PC | 367.9 |
|  | 7days | CC | 340.9 |
| **5** | 24h | PC | 296.4 |
|  | 48h | CC | 329.5 |
|  | 72h | PC | 303.1 |
| **6** | 2h | CC | 205.7 |
|  | 5h | PC | 223.0 |
|  | 24h | PC | 184.5 |
|  | 48h | PC | 226.1 |
|  | 72h | PC | 201.5 |
| **7** | 5h | CC | 267.4 |
|  | 11h | PC | 322.1 |
|  | 48h | PC | 371.7 |
|  | 72h | PC | 349.5 |
| **8** | 5h | CC | 232.7 |
|  | 24h | PC | 272.0 |
|  | 48h | PC | 243.3 |
|  | 72h | PC | 258.6 |
| **9** | 2h | CC | 203.1 |
|  | 5h | PC | 126.2 |
|  | 24h | PC | 99.7 |
|  | 30h | PC | 106.5 |
|  | 48h | PC | 118.6 |
|  | 72h | CC | 145.3 |
| **10** | 5h | PC | 208.4 |
|  | 24h | PC | 203.1 |
|  | 48h | CC | 215.6 |
|  | 72h | PC | 232.7 |
| **11** | 2h | PC | 337.2 |
|  | 5h | CC | 337.2 |
|  | 24h | CC | 267.5 |
|  | 48h | CC | 315.7 |
|  | 72h | PC | 338.9 |
| **12** | 2h | PC | 191.8 |
|  | 5h | PC | 205.2 |
|  | 24h | PC | 224.0 |
|  | 48h | PC | 220.3 |
|  | 72h | CC | 220.0 |
| **13** | 40h | CC | 82.9 |
|  | 46h | CC | 93.7 |
|  | 52h | PC | 107.8 |
|  | 64h | PC | 128.8 |
|  | 70h | CC | 137.8 |
| **14** | 2h | CC | 272.0 |
|  | 5h | PC | 191.8 |
|  | 12h | CC | 162.8 |
|  | 18h | PC | 167.1 |
|  | 24h | CC | 183.0 |
|  | 48h | CC | 281.8 |
|  | 72h | PC | 232.4 |
| **15** | 2h | CC | 331.7 |
|  | 5h | PC | 338.9 |
|  | 24h | CC | 342.8 |
|  | 48h | CC | 282.3 |
|  | 72h | PC | 309.6 |
| **16** | 2h | CC | 212.4 |
|  | 24h | PC | 210.0 |
|  | 48h | PC | 261.1 |
| **17** | 2h | CC | 309.6 |
|  | 5h | PC | 331.7 |
| **18** | 2h | PC | 324.7 |
|  | 24h | CC | 272.0 |
|  | 48h | CC | 236.8 |
|  | 72h | PC | 262.0 |
| **19** | 5h | PC | 164.6 |
|  | 24h | PC | 194.6 |
|  | 48h | PC | 153.3 |
|  | 72h | CC | 203.6 |
| **20** | 2h | PC | 203.4 |
|  | 5h | PC | 167.8 |
|  | 13h | PC | 166.0 |
|  | 24h | PC | 167.9 |
| **21** | 5h | PC | 225.6 |
|  | 48h | PC | 247.0 |
| **22** | 2h | PC | 189.2 |
|  | 5h | PC | 222.4 |
| **23** | 5h | CC | 216.2 |
|  | 72h | PC | 268.2 |
| **24** | 2h | PC | 272.9 |
|  | 5h | CC | 311.7 |
|  | 24h | PC | 290.4 |
|  | 48h | PC | 200.0 |
|  | 96h | PC | 83.3 |
|  | 102h | PC | 93.9 |
|  | 108h | CC | 96.3 |
|  | 114h | PC | 126.5 |
|  | 120h | PC | 126.5 |
|  | 126h | CC | 130.2 |
|  | 138h | PC | 176.8 |
|  | 162h | PC | 216.2 |
| **25** | 2h | CC | 349.7 |
|  | 5h | CC | 323.5 |
|  | 24h | PC | 311.7 |
|  | 48h | PC | 310.3 |
|  | 72h | PC | 311.7 |
| **26** | 5h | CC | 158.7 |
|  | 11h | CC | 138.8 |
|  | 19h | PC | 155.8 |
|  | 43h | PC | 290.4 |
|  | 67h | PC | 358.3 |
| **27** | 2h | CC | 255.0 |
|  | 5h | PC | 311.7 |
| **28** | 8h | UC | 0 |
|  | 14h | UC | 41.0 |
|  | 20h | UC | 82.9 |
|  | 26h | PC | 112.9 |
|  | 32h | PC | 133.0 |
|  | 38h | CC | 135.8 |
|  | 50h | PC | 167.8 |
|  | 74h | PC | 207.4 |
|  | 98h | PC | 226.6 |
| **29** | 2h | PC | 306.1 |
|  | 5h | PC | 290.4 |
|  | 24h | PC | 322.8 |
|  | 48h | PC | 306.1 |
|  | 72h | CC | 310.3 |
| **30** | 2h | PC | 336.1 |
|  | 5h | UC | 310.3 |
|  | 24h | PC | 298.7 |
| **31** | 2h | CC | 350.7 |
|  | 5h | CC | 366.3 |
|  | 24h | CC | 356.9 |
|  | 48h | PC | 366.3 |
|  | 72h | CC | 356.9 |
| **32** | 2h | PC | 280.7 |
|  | 5h | CC | 267.3 |
|  | 48h | PC | 311.7 |
|  | 72h | CC | 238.1 |
|  | 96h | CC | 306.1 |
| **33** | 2h | PC | 416.0 |
|  | 5h | PC | 380.2 |
|  | 24h | CC | 310.3 |
|  | 48h | PC | 494.1 |
|  | 72h | CC | 468.3 |
| **34** | 2h | PC | 356.9 |
|  | 5h | PC | 329.7 |
|  | 24h | PC | 111.8 |
|  | 48h | PC | 90.6 |
|  | 54h | CC | 116.3 |
|  | 66h | PC | 125.6 |
|  | 72h | PC | 154.1 |
| **35** | 24h | PC | 113.6 |
|  | 48h | PC | 117.9 |
|  | 54h | PC | 121.2 |
|  | 72h | PC | 134.4 |
| **36** | 2h | PC | 322.8 |
|  | 5h | PC | 293.2 |
|  | 24h | PC | 316.2 |
|  | 48h | PC | 360.9 |
|  | 72h | PC | 383.0 |
| **38** | 2h | PC | 376.9 |
|  | 5h | PC | 339.1 |
|  | 24h | PC | 195.1 |
|  | 48h | PC | 112.5 |
|  | 72h | PC | 77.0 |
|  | 78h | PC | 81.9 |
|  | 84h | PC | 115.5 |
|  | 90h | PC | 119.5 |
|  | 96h | PC | 146.6 |
|  | 108h | CC | 186.5 |
| **39** | 2h | PC | 167.8 |
|  | 5h | CC | 172.2 |
| **40** | 2h | PC | 282.6 |
|  | 24h | UC | 48.7 |
|  | 30h | PC | 85.7 |
|  | 36h | PC | 109.6 |
|  | 42h | PC | 120.3 |
|  | 54h | PC | 134.4 |
